# Graphene oxide/polydimethylsiloxane composite sponge for removing Pb(ii) from water

**DOI:** 10.1039/d0ra03057k

**Published:** 2020-06-11

**Authors:** Liao Liu, Jiannan Chen, Wuhuan Zhang, Meikun Fan, Zhengjun Gong, Jianqiang Zhang

**Affiliations:** School of Geosciences and Environmental Engineering, Southwest Jiaotong University Chengdu 611756 Sichuan Province China mkfan@swjtu.edu.cn +86 18628194419; Department of Civil, Environmental, and Construction Engineering, University of Central Florida Orlando FL 32816 USA jc6mu@virginia.edu +1 6089605108; Department of Engineering Systems and Environment, University of Virginia Charlottesville VA 22904 USA

## Abstract

An efficient adsorbent to remove Pb(ii) from water was prepared by treating polydimethylsiloxane (PDMS) sponge with polyvinyl alcohol and then coating the sponge with graphene oxide (GO). The GO–PDMS sponge was highly hydrophilic, easily handled during and after use, and easily recycled. The kinetics and isotherms of Pb(ii) sorption onto the GO–PDMS sponge were investigated by performing batch sorption tests. The kinetics of Pb(ii) sorption onto the GO–PDMS sponge indicated that sorption equilibrium occurred rapidly (within 60 min) and that the sorption data could be described using a pseudo-second-order model. Maximum Pb(ii) sorption onto the GO–PDMS sponge occurred at pH > 5. Increasing GO loading on the PDMS sponge increased the amount of Pb(ii) that could be sorbed. The isotherm for Pb(ii) sorption onto the GO–PDMS sponge was non-linear and was well described by the Langmuir isotherm model, indicating that Pb(ii) sorption onto the GO–PDMS sponge was homogeneous and occurred through sorption of a monolayer of Pb(ii). The GO–PDMS sponge, used as a filter, removed Pb(ii) efficiently from water. The Pb(ii) removal efficiencies were more than 50% and the maximum was 85%.

## Abstract 2

A novel sorbent material for Pb(ii) sorption was created by coating graphene oxide (GO) on a pretreated PDMS sponge.

## Graphical abstract

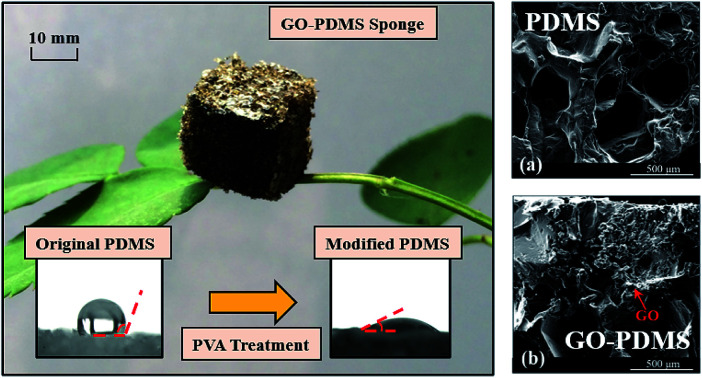

## 1. Introduction

Heavy metal pollution of water negatively affects human health and the environment.^1–3^ Lead (Pb) is one of the most common heavy metals found in wastewater. It is harmful to the human nervous system, blood circulation, and kidneys.^4–6^ Studies of techniques to remove Pb(ii) from wastewater and drinking water have been performed. The most common techniques to remove Pb(ii) from water are coagulation, ion exchange, membrane separation, precipitation, and sorption.^6–12^ Among these techniques, sorption is the most effective because it is efficient, selective, and cost-effective.^7–9^

Graphene-based materials have been used widely as sorbents in water treatment plants, particularly to remove heavy metals.^10,13,14^ Graphene oxide (GO: C_14_OH_42_O_20_) is a derivative of graphene. GO has many oxygen-containing functional groups such as hydroxyl (–OH), carboxyl (–COOH), and epoxy groups. These groups make GO surfaces hydrophilic and negatively charged, meaning GO is an excellent sorbent of positively charged heavy metal ions.^3,15–18^ However, GO generally disperses in water thus it is difficult to collect and recycle.^19^ Three-dimensional (3D) porous sorbents (*e.g.*, sponge-like 3D polydimethylsiloxane (PDMS; (C_2_H_6_OSi)_*n*_) sorbents) that have stable morphologies have been developed and used to treat contaminated water.^20–23^ Sponge-like 3D PDMS sorbents can be easily retrieved and recycled after being used to treat water. In addition, they are highly porous, have high surface areas, and are non-toxic.^24,25^ However, owing to its hydrophobic nature, PDMS can only be used to remove hydrophobic contaminants such as dyes, oil, and organic compounds.^26–33^

A novel approach to preparing a hydrophilic GO–PDMS sponge for removing Pb(ii) ions from aqueous solutions is described in the present study. The GO–PDMS sponge was prepared by dip-coating a PDMS sponge in polyvinyl alcohol (PVA) to modify the surface, followed by loading of GO nanoparticles onto the coated PDMS sponge. The Pb(ii) sorption kinetics and efficiency of the GO–PDMS sponge were evaluated by performing batch tests and the sorption isotherm and sorption mechanism were investigated.

## 2. Materials and methods

### 2.1 Sorbent materials

#### 2.1.1 Fabrication of the hydrophilic PDMS sponge

Commercial PDMS (Sylgard184) (base polymer Sylgard184A and curing agent Sylgard184B) was purchased from Dow Corning (Midland, MI, USA). PDMS sponges were fabricated using the sugar templating method described by Choi *et al.*^26^ and Zhou *et al.*^34^ Briefly, 8 g of the base polymer and 0.8 g of the curing agent were mixed thoroughly in a 25 mL beaker. Then, a sugar cube (18 mm × 17 mm × 9 mm; Tai Gang Food Co., Taizhou, China) was placed in the mixture and the resulting mixture was constantly stirred and kept under vacuum for 2 h. The vacuum was applied to help the PDMS permeate the pores of the sugar cube to form a highly porous PDMS sponge. The mixture was cured in an oven at 80 °C for 3 h, and the sugar cube containing PDMS was sonicated in water for 2 h at 60 °C to dissolve the sugar. The PDMS sponge was then dried at 60 °C overnight.

To modify the surface of the fabricated PDMS sponges, the latter were treated with PVA (molecular weight 145 000, >99% hydrolyzed, Mowiol 28-99; Polysciences, Warrington, PA, USA). The modified PDMS sponge surfaces were hydrophilic, whereas the unmodified PDMS sponge surfaces were hydrophobic. Specifically, the as-prepared PDMS sponge was cleaned with air plasma produced using a YES G1000P system (Alttek Company USA, Huntington Beach, CA, USA) for 15 min to remove hydrophobic functional groups. The PDMS sponge was then immersed in 1% (by weight) PVA solution for 20 min and then dried at 65 °C. This wetting and drying cycle was performed five times.

#### 2.1.2 Preparation of the hydrophilic GO–PDMS sponge

A GO suspension was synthesized using a modified version of the method published by Hummers and Offeman.^35^ Hydrophilic GO–PDMS sponges were prepared using the dip-coating method described by Zhou *et al.* and Liang *et al.*, respectively.^34,36^ Each PVA-modified PDMS (PPDMS) sponge was immersed in 5 mg mL^−1^ GO solution under vacuum for 30 min. The GO penetrated the porous PDMS structure through capillary action. The GO–PDMS sponge was then dried at 65 °C for 45 min. The wetting and drying cycle was repeated three times.

#### 2.1.3 Characterization of the PDMS and GO–PDMS sponge

The Brunauer–Emmett–Teller surface areas and average pore diameters of the PDMS and GO–PDMS sponges were measured *via* N_2_ physisorption at −195.8 °C on a Micromeritics ASAP 2020 system. The surface area of the PDMS sponge was 16.3 m^2^ g^−1^, whereas that of the GO–PDMS sponge was slightly higher (19.2 m^2^ g^−1^) owing to the GO coating. The average pore diameters of the PDMS and GO–PDMS sponges were 4.2 and 3.5 nm, respectively. The micro-structures of the PPDMS and GO–PDMS sponges were imaged using an Inspect F scanning electron microscope equipped with an energy-dispersive X-ray spectroscopy unit (FEI, Hillsboro, OR, USA). The functional groups in the PPDMS and GO–PDMS sponges were analyzed using a Spectrum Two Fourier-transform infrared spectrometer (PerkinElmer, Waltham, MA, USA). The analyses were performed using KBr disks and within a wavenumber range of 400–4000 cm^−1^. The hydrophobicity of the original PDMS and PPDMS sponges was characterized by determining the water contact angles. A 2 μL droplet of deionized water was dripped onto the surface of a sponge, and the contact angle was measured using an XED-SPJ contact angle goniometer (Hake, Beijing, China). The zeta potential of the GO–PDMS sponge was measured at 20 ± 1 °C using a zeta potential analyzer (SurPASS 2, Anton Paar, Graz, Austria). The GO–PDMS sponge was conditioned in 1 mM KCl solution at final pHs of 2.5–6.0 for 24 h in a shaking bath. The pH of the suspension was adjusted using 50 mM HCl and 50 mM NaOH.

### 2.2 Batch sorption tests

#### 2.2.1 Kinetics and isotherms for Pb(ii) sorption onto the GO–PDMS sponge

The effects of contact time, solution pH, and GO loading on the kinetics of Pb(ii) sorption onto the GO–PDMS sponges were evaluated by performing batch sorption tests. The experiments were performed in aqueous solutions prepared by dissolving reagent-grade Pb(NO_3_)_2_ in ASTM Type II deionized water. Then, 0.1 g of the GO–PDMS sponge (prepared using 5 mg mL^−1^ GO solution) was mixed with 10 mL of a Pb(ii) solution (to achieve a solid-to-liquid ratio of 1 : 100) in a polypropylene centrifuge tube. The centrifuge tube was kept at 30 °C and shaken at 150 rpm for a specified contact time. Batch sorption tests were performed using contact times between 5 and 120 min, at Pb(ii) concentrations of 10 and 20 mg L^−1^, and pH 5.0 ± 0.1. Following determination of the contact time required to reach adsorption equilibrium, the effect of pH on the sorption equilibrium was assessed by performing tests using a Pb(ii) concentration of 50 mg L^−1^ and pH values between 2.5 and 6.0. The pH was adjusted by adding 0.1 M NaOH or 0.1 M HCl. The GO–PDMS sponges used in these tests were prepared using a 5 mg mL^−1^ GO solution, but the effect of GO loading on Pb(ii) sorption was investigated using GO–PDMS sponges prepared using GO concentrations between 1 and 5 mg mL^−1^.

Data to allow Pb(ii) sorption isotherms for the GO–PDMS sponges prepared using 5 mg mL^−1^ GO solution to be drawn were acquired by performing tests at pH 5.0 ± 0.1 using Pb(ii) concentrations of 5–80 mg L^−1^ and determining the Pb(ii) concentrations at equilibrium. Each batch sorption test result is the mean of triplicate tests, and the error for each test was less than 5%.

#### 2.2.2 Filtration experiments

The GO–PDMS sponge was easily handled and recycled. Thus, it was expected that it could be used as a water filter to remove Pb(ii) from water. Filtration experiments simulating the use of GO–PDMS sponges as water filters were performed. For the studies, 0.1 g of the GO–PDMS sponge was placed in a 5 mL polypropylene syringe, then 10 mL of a solution containing Pb(ii) at concentrations between 1 and 20 mg L^−1^ was passed through the GO–PDMS sponge 10 times. The Pb(ii) concentration in the filtrate was then determined.

### 2.3 Solid and water chemistry analysis

Elemental analyses of the GO–PDMS sponges before and after the Pb(ii) sorption tests were performed using an ESCALAB 250Xi X-ray photoelectron spectroscopy (XPS) instrument (Thermo Fisher Scientific, Waltham, MA, USA). The solid and liquid in a given mixture after a batch or filtration experiment were separated by centrifuging the mixture at 4000 rpm for 20 min, then the supernatant was transferred to a 15 mL polypropylene centrifuge tube for chemical analysis. The initial and final Pb(ii) concentrations were determined using a Hitachi Z-5000 atomic absorption spectrometer (Hitachi High-Technologies, Tokyo, Japan). Control tests were performed in the absence of GO–PDMS to assess the potential sorption of Pb(ii) onto the centrifuge tubes or other equipment during the test process. The results confirmed that negligible Pb(ii) sorption occurred onto the propylene centrifuge tubes and other equipment.

## 3. Results and discussion

### 3.1 Morphology and surface characteristics of the GO–PDMS sponge

**Fig. 1:**
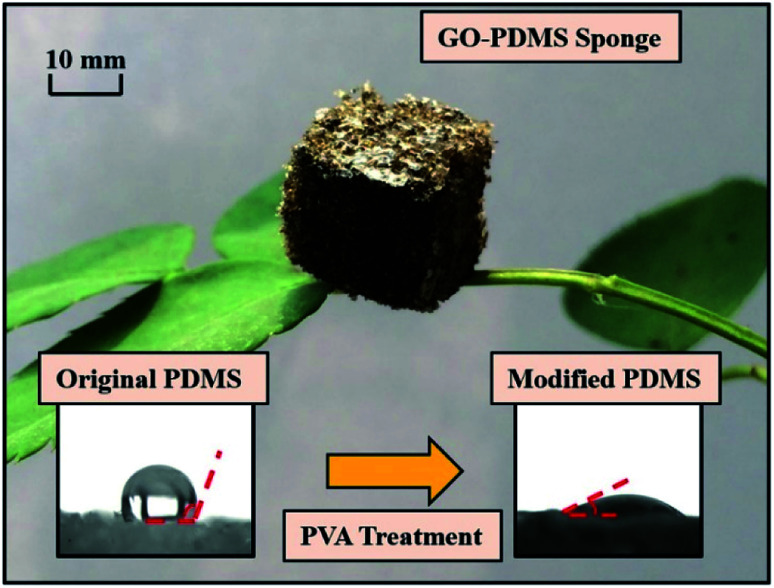
Lightweight graphene oxide (GO) polydimethylsiloxane (PDMS) sponge supported by a leaf, and the contact angles of a 2 μL water droplet on the surface of a PDMS sponge (114.7°) and the surface of a PDMS sponge modified with polyvinyl alcohol (PVA) (26.4°).

**Fig. 2:**
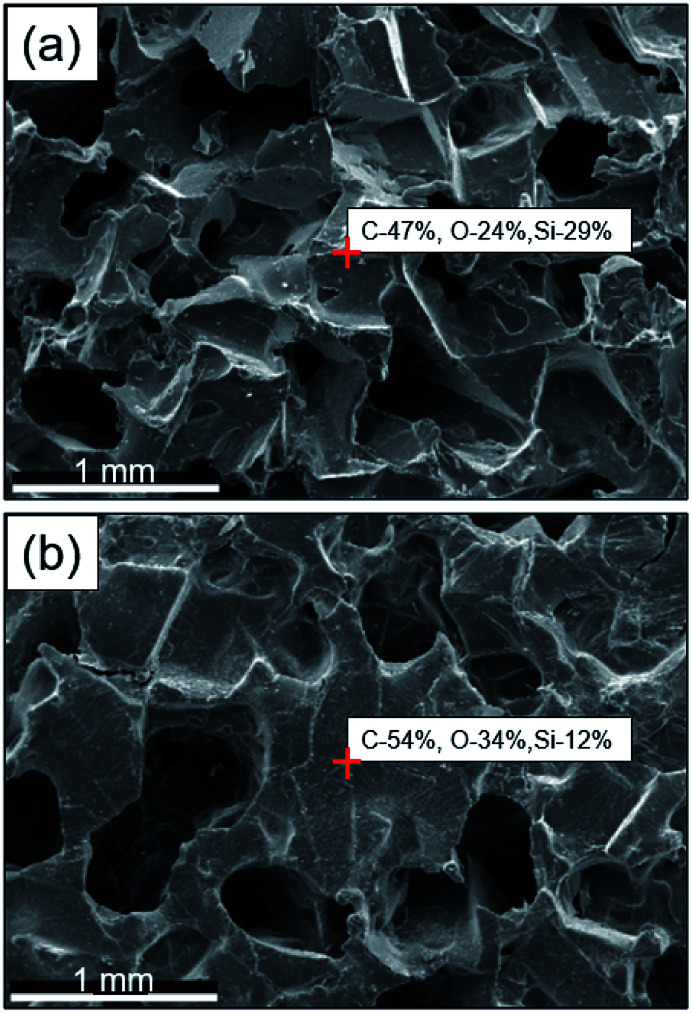
Scanning electron microscopy images of (a) the network structure of the polydimethylsiloxane sponge modified with polyvinyl alcohol and (b) the graphene oxide-coated network structure of the polydimethylsiloxane sponge modified with polyvinyl alcohol.

The GO–PDMS sponge was a light material with a 3D porous structure and it was easily supported by a leaf (Fig. 1). The 3D network structure was obtained using the sugar templating method. Scanning electron microscopy images of PPDMS and GO–PDMS sponges indicated that both types of sponge had porous micro-structures. The PPDMS sponge had smooth surfaces and distinct pores (Fig. 2a) but loading of GO particles onto the PPDMS sponge caused the sponge surfaces to become rough and the pores filled with GO (Fig. 2b). Additionally, the loading of GO altered the chemical composition of the sponge, resulting in a higher C content (54%) and a lower Si content (12%) in the GO–PDMS when compared with those in PDMS (47% and 29%, respectively). The PPDMS substrate could easily take on different shapes while maintaining excellent morphological integrity, meaning the GO–PDMS sponge could easily be recycled. The hydrophobicity of the original PDMS was changed by the PVA modification, which introduced hydrophilic oxygen-containing functional groups to the surfaces (Fig. 1). The original PDMS sponge (before PVA modification) had a water contact angle of 114.7° and a hydrophobic surface. PVA modification allowed water droplets to spread rapidly and penetrate the PPDMS sponge. The PPDMS sponge was hydrophilic and the water contact angle was 26.4°. The hydrophilicity and porous structure of the GO–PDMS sponge gave the material a high surface area (19.2 m^2^ g^−1^) that could sorb dissolved Pb(ii).

**Fig. 3:**
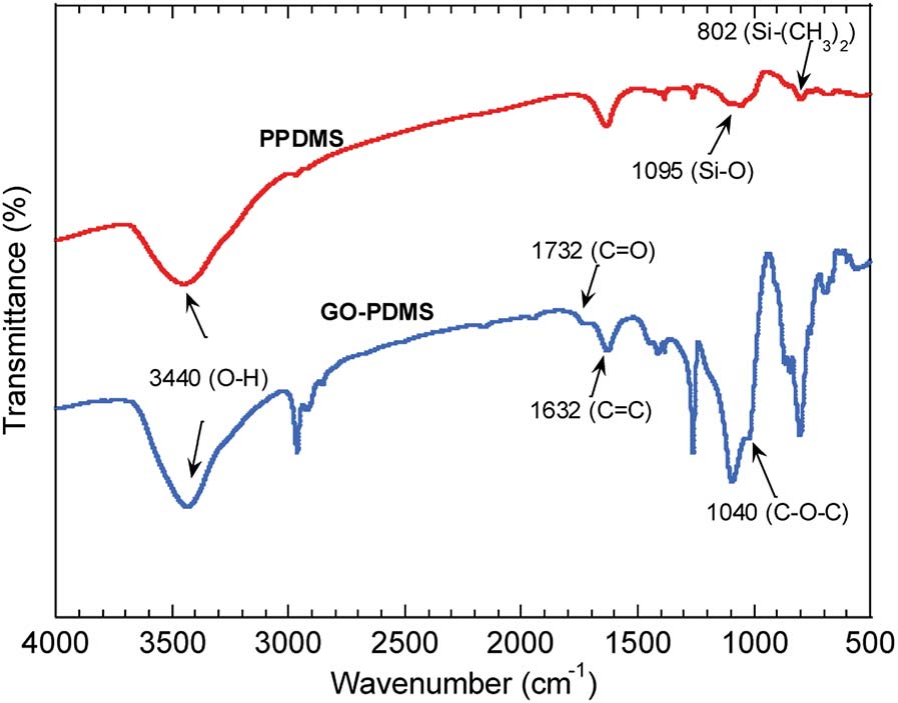
Fourier-transform infrared spectra of the polydimethylsiloxane sponge modified with polyvinyl alcohol (PPDMS) and PPDMS modified with graphene oxide (GO–PDMS).

The Fourier-transform infrared spectra of PPDMS and GO–PDMS sponges indicated the presence of abundant hydroxyl groups (O–H band at 3440 cm^−1^), thereby indicating that PVA modification caused the PDMS sponge surface to become hydrophilic (Fig. 3). Specifically, the Fourier-transform infrared spectrum of PPDMS displayed three major stretching vibration bands at 3440, 1095, and 802 cm^−1^, which can be attributed to the presence of O–H, Si–O, and Si–(CH_3_)_2_ functional groups, respectively.^37^ Introducing GO resulted in the appearance of stretching vibration bands at 1732, 1623, and 1040 cm^−1^, corresponding to C=O, C=C, and C–O–C groups.^18,38–40^ These functional groups are present in GO.^36^ The adsorption of adsorbates onto GO–PDMS is expected to proceed *via* chelation reactions, involving the oxygen-containing functional groups, *e.g.*, C=O and C–O–C.^41,42^

### 3.2 Kinetics of Pb(ii) sorption onto the GO–PDMS sponge

#### 3.2.1 Effect of contact time on Pb(ii) sorption

**Fig. 4:**
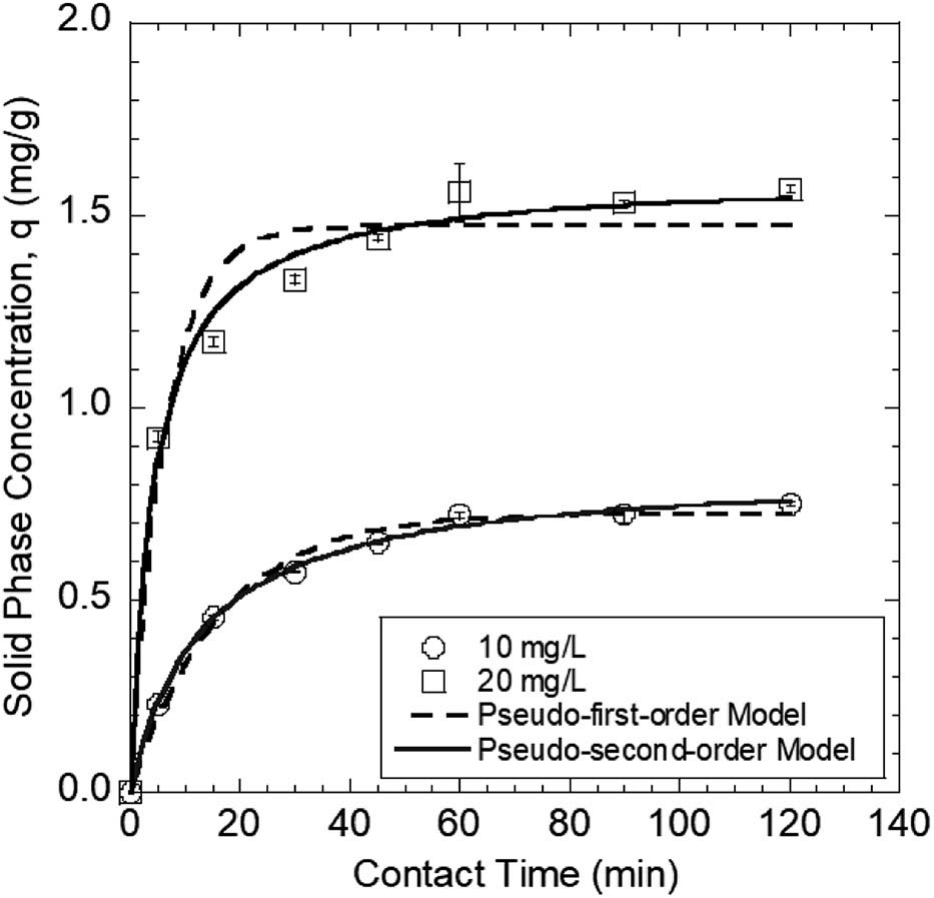
Sorption of Pb(ii) onto the polydimethylsiloxane sponge modified with polyvinyl alcohol and graphene oxide over time at different Pb(ii) solution concentrations of 10 and 20 mg L^−1^. Error bars represent standard deviations from triplicate tests.

The contact time is a key parameter affecting whether heavy metal sorption reaches equilibrium.^43^ Changes in the amount of Pb(ii) sorbed onto the GO–PDMS sponges exposed to 10 and 20 mg L^−1^ Pb(ii) solutions as a function of time (120 min in total) are shown in Fig. 4. The time taken for Pb(ii) sorption onto the GO–PDMS sponge to reach equilibrium was less than 120 min (*i.e.*, between 24.2 and 81.2 min depending on the model). The Pb(ii) concentrations in the GO–PDMS sponges exposed to 10 and 20 mg L^−1^ Pb(ii) solutions at equilibrium (determined after 120 min of exposure) were 0.75 and 1.6 mg g^−1^, respectively. The time-dependence of Pb(ii) sorption was described using the pseudo-first-order model shown in eqn (1) and the pseudo-second-order model shown in eqn (2).

1 ln(*q*_e_ − *q*_*t*_) = ln *q*_e_ − *k*_1_*t*

2
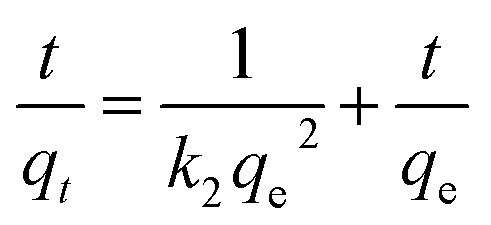
In eqn (1) and (2), *q*_*t*_ and *q*_e_ are the amounts of Pb(ii) sorbed onto the GO–PDMS at time *t* and equilibrium, respectively, and *k*_1_ and *k*_2_ are the sorption rate constants. The *q*_e_ values and sorption rate constants are shown in Table 1. The time-dependence of Pb(ii) sorption was described better by the pseudo-second-order model than the pseudo-first-order model, as indicated by the higher *R*_2_ value obtained for the pseudo-second order model-fitted data. The batch tests described subsequently were all conducted for 120 min to ensure that equilibrium was reached.

**Table 1:** Kinetic parameters of pseudo-first order and pseudo-second order models for Pb(ii) sorption onto GO–PDMS

Original Pb(ii) concentration (mg L^−1^)	*q*_e,exp_ a (mg g^−1^)	Pseudo-first-order				Pseudo-second order			
		*q*_e,cal_ b (mg g^−1^)	*k*_1_ (min^−1^)	*t*_e_ c (min)	*R* ^2^	*q*_e,cal_ (mg g^−1^)	*k*_2_ (g mg^−1^ min^−1^)	*t*_e_ (min)	*R* ^2^
10	0.75	0.72	0.062	24.6	0.99	0.84	0.092	81.2	1.0
20	1.57	1.47	0.16	63.1	0.96	1.60	0.15	71.5	0.95

a *q*_e,exp_, solid-phase concentration at equilibrium determined experimentally.

b *q*_e,cal_, calculated solid-phase concentration at equilibrium.

c *t*_e_ = time to reach equilibrium.

#### 3.2.2 Effect of pH on Pb(ii) sorption

**Fig. 5:**
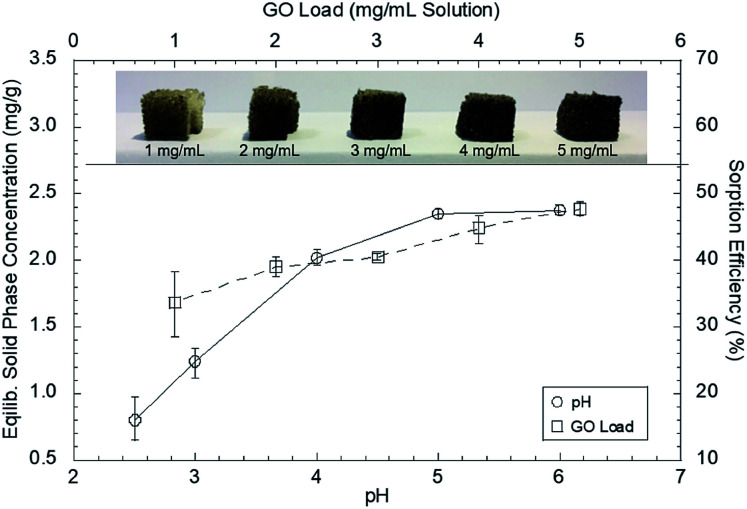
Effect of pH and graphene oxide (GO) loading on the sorption capacity of the GO polydimethylsiloxane sponge for Pb(ii) ions (initial Pb(ii) concentration is 50 mg L^−1^). Error bars represent standard deviations from triplicate tests.

The pH is another key factor that influences both the adsorption and desorption of heavy metals from a sorbent.^43,44^ If the pH is higher than the point of zero charge (PZC) of the sorbent material, the sorbent will attract dissolved cations such as Pb(ii).^45^ The sorption capacity of the GO–PDMS sponge varied considerably over the pH range of 2.5–6.0 (Fig. 5). The sorption efficiency (SE), as described in eqn (3), and the *q*_e_, as described in eqn (4), were determined:3
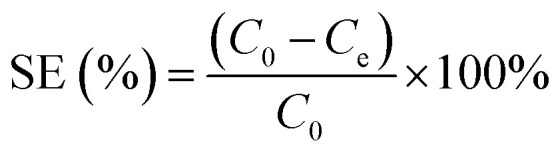

4 *q*_e_ (mg g^−1^) = (*C*_0_ − *C*_e_)*V*/*m*

where *C*_0_ is the initial Pb(ii) concentration in solution, *C*_e_ is the Pb(ii) concentration in solution at equilibrium, *V* is the volume of the solution, and *m* is the mass of the GO–PDMS sponge.

The Pb(ii) concentration in the solid phase and the sorption efficiency increased as the solution pH increased from 2 to 6 (Fig. 5). The PZC of the GO–PDMS sponge was determined to be pH 3.3 from zeta potential measurements. At low pH values (<3.3), H^+^ is abundantly present on the GO–PDMS surface, resulting in a positively charged surface. This surface results in coulombic repulsion of heavy metal cations, thereby limiting the sorption capacity of GO–PDMS. When the pH is increased, the repulsion weakens, thus increasing the sorption capacity of the GO–PDMS. The maximum Pb(ii) concentration in the solid phase (2.4 mg g^−1^) and the maximum sorption efficiency (47%) were observed at pH values above 5. Precipitation of Pb(ii) occurred at pH 6, probably because the solution became saturated with cerussite (PbCO_3_) and hydrocerussite (Pb_3_(CO_3_)_2_(OH)_2_).^46,47^ As Pb(ii) precipitation interfered with the sorption test results, experiments were not performed at pH values above 6. The subsequent batch experiments were thus performed at pH 5.0 ± 0.1 to avoid the onset of Pb(ii) precipitation.

#### 3.2.3 Effect of the GO load on Pb(ii) sorption

The sponges became darker as the GO solution concentration increased, indicating that increasing the GO solution concentration gave a higher GO load on the PDMS sponge surfaces (Fig. 5). The Pb(ii) concentration in the GO–PDMS sponge solid phase at equilibrium increased from 1.7 to 2.4 mg g^−1^ as the GO load increased. The sorption efficiency increased from 37.7% to 47.7% as the GO load increased. The Pb(ii) concentration in the solid phase and the sorption efficiency increased as the GO load increased likely because the number of active functional groups (*e.g.*, carboxyl and hydroxyl groups) provided by GO on the PDMS sponge surfaces would increase as the GO load increased. Similar results were found in a study performed by Zhang *et al.* and Anfar *et al.*^48–50^

### 3.3 Isotherms for Pb(ii) sorption onto the GO–PDMS sponge

**Fig. 6:**
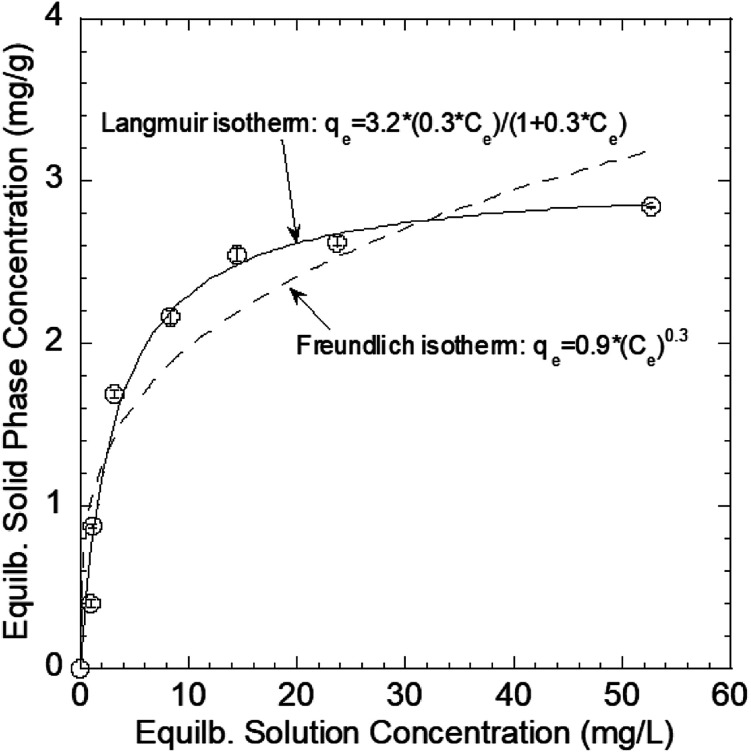
Langmuir and Freundlich sorption isotherm models for the sorption of Pb(ii) onto the graphene oxide polydimethylsiloxane sponge. Error bars represent standard deviations from triplicate tests.

The Pb(ii) sorption isotherms for the GO–PDMS sponges were non-linear. Langmuir and Freundlich sorption isotherm models were fitted to the data, and the results are shown in Fig. 6. These non-linear models were used to evaluate the sorption process and identify the mechanism through which Pb(ii) sorbed onto the GO–PDMS sponge. The Langmuir isotherm assumes that the surface of the sorbent is uniform, monolayer sorption of the sorbate occurs, there are a finite number of sorption sites, and sorption occurs with the same sorption energy at all sites. The Freundlich isotherm assumes that large numbers and various types of sorption sites act simultaneously. The Freundlich isotherm is suitable for nonideal sorption onto heterogeneous surfaces with various free energies of sorption. The Langmuir sorption isotherm model and Freundlich sorption isotherm model can be expressed as shown in eqn (5) and (6), respectively.5
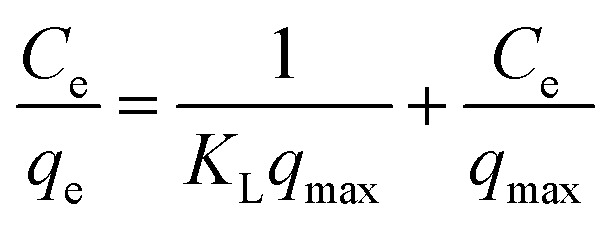
6
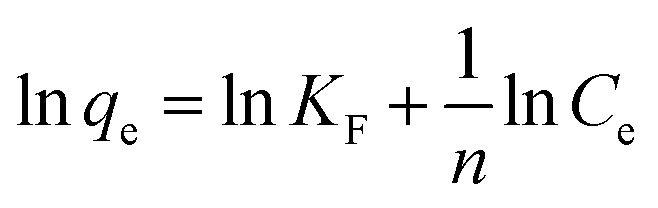
*K*_L_ is the Langmuir equilibrium constant, *q*_max_ is the maximum sorption capacity, and *K*_F_ and *n* are the Freundlich parameters. The fitting parameters for the Langmuir and Freundlich sorption isotherm models are shown in Table 2. The Pb(ii) sorption isotherm for the GO–PDMS sponge was described better by the Langmuir isotherm (*R*^2^ = 0.98) than the Freundlich isotherm (*R*^2^ = 0.90), indicating that Pb(ii) sorption onto the GO–PDMS sponge occurred through monolayer sorption onto homogeneous sorption sites. The *K*_L_ value was 0.3 (*i.e.*, between 0 and 1), implying that Langmuir sorption was favored.

**Table 2:** Fitting parameters of the Langmuir and Freundlich models for sorption of Pb^2+^ onto GO–PDMS

Langmuir fitting parameters		Freundlich fitting parameters	
*q*_max_ (mg g^−1^)	3.2	*K*_F_ [(mg g^−1^) (L mg^−1^)^1/*n*^]	0.9
*K*_L_ (L mg^−1^)	0.3	1/*n*	0.3
*R* ^2^	0.98	*R* ^2^	0.90

**Fig. 7:**
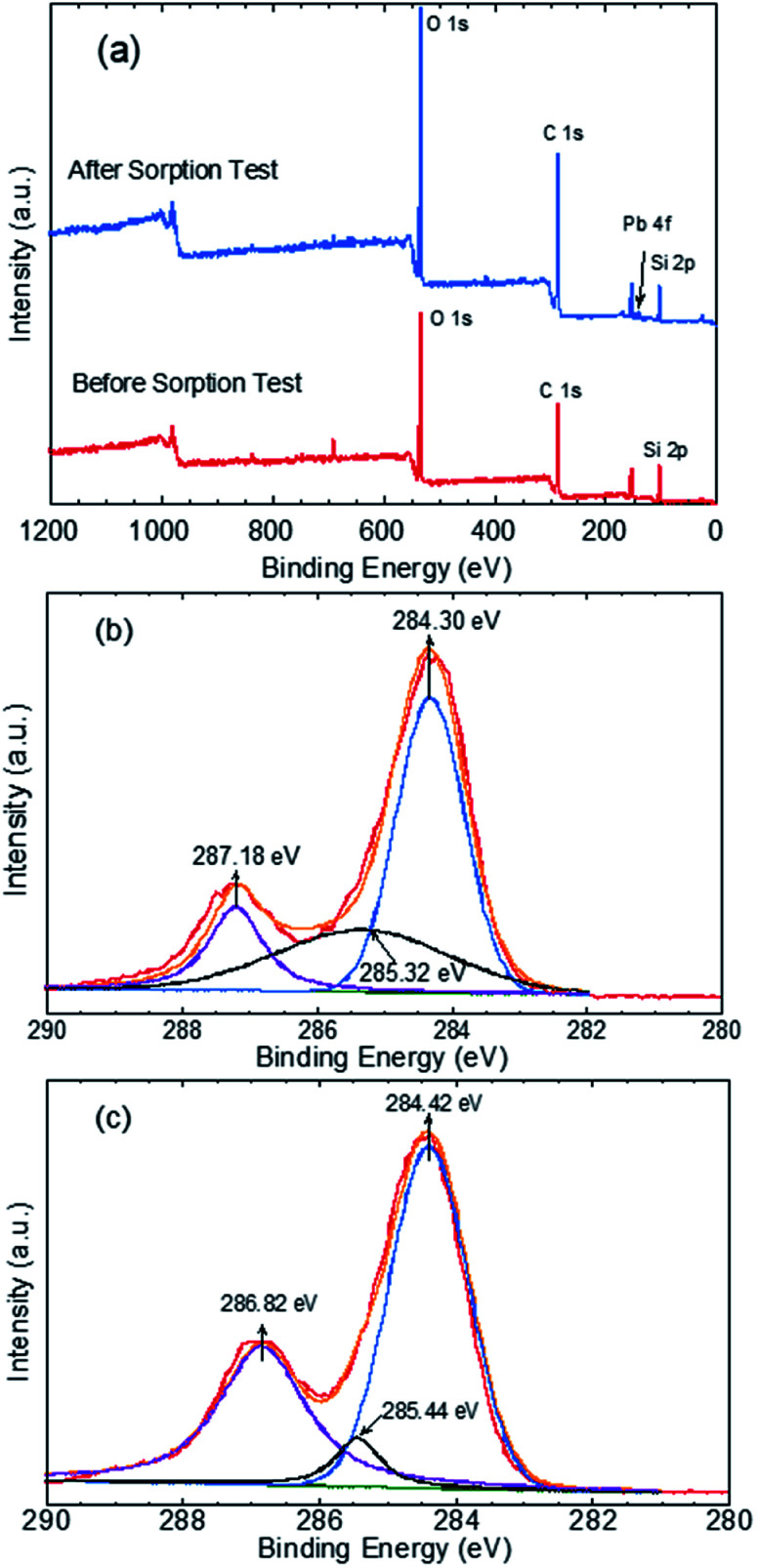
X-ray photoelectron spectra of graphene oxide polydimethylsiloxane sponges before and after being used in a Pb(ii) sorption test. (a) Wide-scan spectra of the sponges before and after Pb(ii) sorption. Deconvoluted C 1s peaks (b) before and (c) after Pb(ii) sorption.

The mechanism through which Pb(ii) sorbed onto the GO–PDMS sponge was evaluated by XPS to identify the elements and functional groups on the GO–PDMS sponge surface before and after sorption. Specifically, a GO–PDMS sponge that had been exposed to 80 mg L^−1^ Pb(ii) solution and a GO–PDMS sponge that had not been exposed to Pb(ii) solution were analyzed. The wide-scan spectra and the characteristic peaks before and after Pb(ii) sorption are shown in Fig. 7—O 1s, C 1s, and Si 2p peaks were observed for both sponges, and these peaks reflected the elemental components of PDMS and GO. In addition, a Pb 4f peak was detected in the sponge that was exposed to Pb(ii) solution, indicating the sorption of Pb(ii) onto the GO–PDMS sponge. The deconvoluted C 1s peaks for both sponges are shown in Fig. 7b and c. Before sorption, the XPS pattern of GO–PDMS displayed three peaks at binding energies of 284.30, 285.32, and 287.18 eV that were assigned to C–C, C–O, and C=O bonds, respectively.^21,23^ After the sorption tests, the binding energy of C=O bond shifted to 286.82 eV, suggesting that oxygen-containing groups in GO (*e.g.*, alcohol and carboxylate groups) were actively involved in the sorption reactions. Slight shifts in the binding energies of C–C and C–O bonds (to 284.42 and 285.44 eV, respectively) were also detected, indicating that the C=O functional group plays a prominent role in the sorption of Pb(ii) onto the GO–PDMS sponge. This observation further proves the better fitting of the sorption isotherms using the Langmuir isotherm model when compared with the Freundlich isotherm model, as the former model is based on the assumption of a surface containing a finite number of uniform sorption sites (*i.e.*, carboxylate groups in the present study).

### 3.4 Filtration experiments

**Fig. 8:**
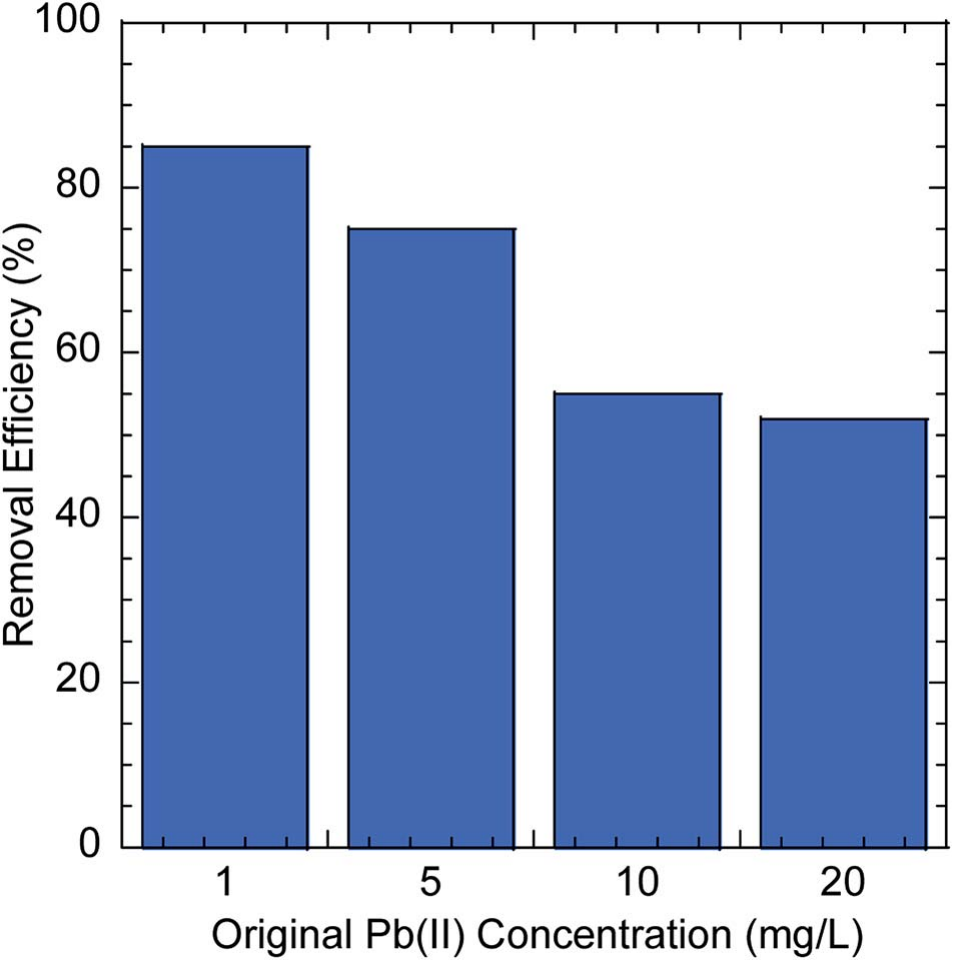
Pb(ii) removal efficiencies obtained when graphene oxide polydimethylsiloxane sponges were used to filter solutions containing Pb(ii) at different concentrations.

The possibility of using GO–PDMS sponges to filter water to remove Pb(ii) was evaluated. The simple handling and recycling of GO–PDMS sponges would be convenient when filtering water. The Pb(ii) removal efficiencies achieved using GO–PDMS sponges to treat solutions containing Pb(ii) at concentrations between 1 and 20 mg L^−1^ are shown in Fig. 8. When a GO–PDMS sponge was used to treat a 1 mg L^−1^ Pb(ii) solution, 85% of the Pb(ii) was removed after 10 filtering cycles. The removal efficiency decreased as the Pb(ii) concentration in solution increased, but a rather high removal efficiency of >50% was still attained when the Pb(ii) concentration in solution was 20 mg L^−1^. These results indicated that the GO–PDMS sponge is an ideal material for removing Pb(ii) from water.

## 4. Conclusions

An efficient sorbent to sorb Pb(ii) from water was synthesized by treating a PVA-modified PDMS sponge with GO. The aim was to develop a cost-effective method for removing Pb(ii) from water using GO in a material that is easy to handle and recycle. The PDMS sponge was hydrophobic, but PVA modification made the sponge surface hydrophilic. The kinetics and isotherms of Pb(ii) sorption onto the GO–PDMS sponge were evaluated by performing batch sorption tests. The conclusions below were drawn from the results.

■ The kinetics of Pb(ii) sorption onto the GO–PDMS sponge indicated that equilibrium was reached within 60 min. The data were described well using a pseudo-second-order model. Maximum Pb(ii) sorption onto the GO–PDMS sponge was found at pH > 5. Increasing the GO loading on the PDMS sponge increased the Pb(ii) sorption capacity of the GO–PDMS sponge.

■ The Pb(ii) sorption isotherm for the GO–PDMS sponge was non-linear and fitted well using the Langmuir isotherm model. Pb(ii) sorbed onto the GO–PDMS sponge as a homogeneous monolayer.

■ The GO–PDMS sponge can be used to filter water to remove Pb(ii) and is easily handled and disposed of. A Pb(ii) removal efficiency of up to 85% was achieved when the GO–PDMS sponge was used to filter a dilute Pb(ii) solution (1 mg L^−1^) and a Pb(ii) removal efficiency of 50% was achieved when the GO–PDMS sponge was used to filter a more concentrated solution (20 mg L^−1^).

## Conflicts of interest

There are no conflicts to declare.

## Supplementary Material
